# Identifying Key Factors Associated with Green Justice in Accessibility: A Gradient Boosting Decision Tree Analysis

**DOI:** 10.3390/ijerph191610357

**Published:** 2022-08-19

**Authors:** Sainan Du, Huagui He, Yanfang Liu, Lijun Xing

**Affiliations:** 1 Faculty of Resources and Environmental Science, Hubei University, 368 Youyi Road, Wuhan 430062, China; 2 Guangzhou Urban Planning and Design Survey Research Institute, Guangzhou 510060, China; 3 School of Resource and Environment Science, Wuhan University, 129 Luoyu Road, Wuhan 430079, China

**Keywords:** park green space (PGS), green justice, accessibility and equity, gradient boosting decision tree (GBDT), nonlinear influence, Wuhan

## Abstract

Park green space (PGS) provides numerous environmental and health benefits for urban residents, and raises the issue of green justice for its uneven distribution in cities. Previous studies focus more on the measurements of spatial equity in accessibility, but are limited in exploring its impacts—especially the nonlinear influence. This study first measures accessibility and equity in two traffic modes, and then explores the nonlinear influence of multidimensional factors by using the gradient boosting decision tree (GBDT) model across the central urban area of Wuhan. The results show significant spatial disparities in spatial accessibility and equity by walking and driving within 15 min. Multidimensional factors—including characteristics of PGS, the built environment, and socioeconomic factors—present stronger nonlinear influences on spatial accessibility and equity, and the nonlinear influence indicates that the contributions of the built environment and socioeconomic factors are greater than those of park characteristics, accounting for at least 79.76%. The key variables affecting the accessibility and equity are not completely consistent, leading to synergistic and heterogeneous effects, which may provide policy implications for streets where accessibility and equity are mismatched. These findings could provide guidance for PGS planning by decision-makers to improve the living environment and urban health.

## 1. Introduction

Urban green space is an important link between residents and nature, especially in the form of urban parks [1]. It promotes public health, protects the urban ecological environment, and enhances residents’ happiness by reducing and mitigating the negative impacts of urbanization [2,3]. In particular, urban parks improve mental health and wellbeing by providing opportunities for physical activity and reducing stress [4]. Thus, providing residents with sufficient and equitable park green space (PGS) is a common goal of urban planners [5,6]. However, with the continuous expansion of urban construction land and populations, green space is becoming less and less, resulting in a serious shortage of green space [7]. Therefore, the research on green justice can make a positive contribution to the issue of urban green space and mental health.

Owing to the growing concern with green justice, providing adequate and equitable green space for residents has become an important goal for urban planners [3,8]. The unfair usage and uneven distribution of green space has become prominent with the rapid development of urbanization in developed or developing countries. Therefore, the primary focus of green justice is the equity and justice of the usage and distribution of green space [9,10,11]. An increasing number of studies turn to measuring the equality of opportunities for access to green spaces of different types for urban residents in a given region, contributing to the research on green justice [3,8,11]. Research on spatial equity of urban parks mainly examines three stages: regional equality, spatial equity, and social equity. Among them, the spatial equity stage emphasizes the utilization efficiency and spatial allocation of land resources, which are usually measured by per capita green space index and accessibility [5]. The spatial mismatch between supply and demand of park services in a geographic unit is also measured in this stage [12]. In the social equity stage, the focus shifts from local equity to human equity, e.g., the equity of service supply between different social groups [8,13], demand indices [14], Nicky coefficients, and Lorenz curves [15,16]. Studies show that the supply of green spaces produces disparities due to factors such as income, race, age, gender, and power capital [17]. Based on the above research topics, the measurements of spatial equity have become the key aspect of the research.

Accessibility is considered to be an effective indicator of spatial equity, and can be measured in many ways, such as buffer analysis [18], kernel density estimation [19], network-constrained service area methods [20], the Thiessen polygons method [21], and gravity-based models [22]. The two-step floating catchment area (2SFCA) method and its improved form, as a dichotomous technique of gravity-based models, are currently regarded as a suitable approach, and have been widely used to measure potential spatial access to PGS [23], because they can consider both the supply and demand of parks under the reality of road networks [16]. Moreover, comprehensive perspective parameters (e.g., park proximity, acreage, and quality) have been highlighted in green justice studies to measure spatial access to parks multidimensionally [24]. The Gini coefficient is another index for measuring equity, but it is more applicable to large-scale research than on studies of communities or living quarters [25,26]. Abundant empirical evidence using these indicators can be found in cities from different countries, but the results vary due to green space definitions, scales, and political and geographical characteristics.

To guide the planning practice better, researchers have attempted to adjust the influencing factors causing the disparity in accessibility to improve the equity of green space. For instance, studies have linked the accessibility or the spatial differences of green provision to the socioeconomic status and demographic factors of different social groups, such as ethnic or religious characteristics [27], immigration status [28], age [16], and personal ability [14,29]. Scholars have suggested that the built environment presents a certain impact on spatial accessibility, including community size and green assets of the community [30], the distance of urban parks, and the richness of trees [31]. Other scholars associate the complexity of social and economic conditions with accessibility and equity [32,33]. Moreover, the reasons for spatial differences in supply and demand of urban parks vary in practice, but maximizing accessibility and minimizing social and spatial inequality caused by accessibility (or lack thereof) should be the common goals of urban planners [34]. Therefore, exploring the impact mechanism on green justice may effectively help urban planners to implement concrete measures to reduce the inequality of green supply and allocation.

Although accessibility is an effective indicator of equity, it only represents the opportunity of access to park services for residents. Improving accessibility may not improve the equity of areas in need of planning [35]. The key factors that affect accessibility are not necessarily important factors that affect fairness. Thus, the influencing factors of accessibility and equity must both be explored, and the common elements necessary to provide effective guidance for urban planning under the objective of equity must be determined. Most previous studies focus on the linear relationships between spatial accessibility and the influencing factors, but they are limited in exploring the nonlinear relationships [28,30]. Although the measurement of linear relationships can identify factors related to accessibility, the mechanisms of their influence on accessibility are unclear, which brings confusion to the adjustment of planning policy.

To alleviate these gaps, we adopted the GBDT model to explore the nonlinear influence of multidimensional factors on spatial accessibility and equity. We also committed to improving the level of green justice from the perspective of urban planning based on the analysis of synergy and heterogeneity of influencing factors. Specifically, this study aimed to (1) measure the accessibility and equity of PGSs by using the Gaussian-based 2SFCA method; (2) examine the important factors and identify the key variables in spatial accessibility and equity by using the GBDT model, including PGSs’ characteristics, the built environment, and socioeconomic factors; and (3) analyze the synergistic and heterogeneous effects on accessibility and equity for future policy decisions and planning. The achievement of the aforementioned objectives could help address two questions: (1) What are the key variables that present nonlinear influences on the spatial accessibility and equity of PGSs? (2) Do these variables have synergistic and heterogeneous effects, and what enlightenment can they bring to urban planning?

## 2. Materials and Methods

### 2.1. Study Area and Data

**Figure 1:**
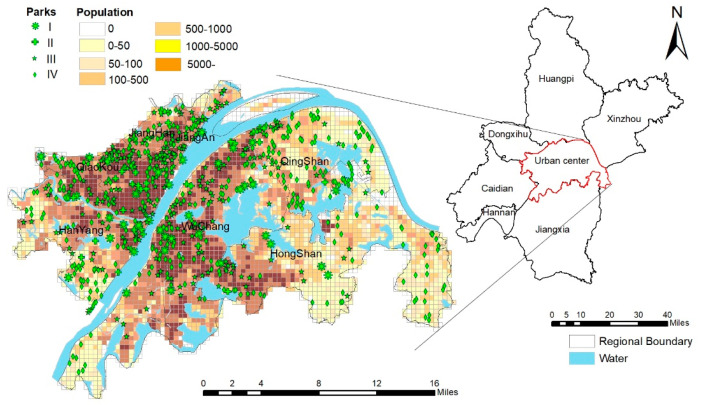
Spatial distribution of PGSs and population of Wuhan’s central district.

Wuhan is located in the central reaches of the Yangtze River. It is the capital city of Hubei Province, and is one of the six major cities in Central China. Wuhan’s urban center was taken as the research area, which includes Jiangan Jianghan, Qiaokou, Hanyang, Wuchang, Hongshan, and Qingshan. The central urban area is 955.15 km^2^, the permanent population is 6.7588 million, and the average population density is 0.708 million/km^2^. A total of 698 parks was included in our study until 2020. Among them, 36 comprehensive parks, 31 theme parks, 236 community parks, and 395 amusement parks have been constructed in Wuhan’s urban center for residents to enjoy the parks’ green services (see Figure 1). On the whole, the per capita park green space reaches 7.35 m^2^/per person, and the spatial distribution of parks is concentrated along the Yangtze River and the Han River.

Our study measured the accessibility of PGSs in 500 × 500 grid scales, and calculated the average accessibility and equity in street or district scales. The basic data were provided by relevant administrative government departments of Wuhan—(1) the urban land-use types and road data were provided by the Wuhan Land Management, Planning Department, and Transport agency in 2020; (2) the Wuhan Statistics Bureau provided data on residential population, GDP, social fixed-asset investment, retail sales of social consumer goods, and budget revenue in 2020. PGSs were divided into four types: comprehensive parks, theme parks, community parks, and amusement parks. This division takes the Classification Standard of Urban Green Space (CJJ/T85-2017) as a reference. We obtained the attribute data of PGSs for 2020—including location, area, and type—from the world map and the Wuhan City Park Directory. Furthermore, the demographic data in each grid were obtained by apportioning the building area to each grid. The building area data used for apportionment came from the building area data of the Wuhan Institute of Surveying and Mapping, and the park entrances were identified through a variety of historical data and online maps. Additionally, we used road intersections as supply points to identify the small community parks and roadside green spaces.

### 2.2. Variables

In the process of selecting influencing variables, we combined the existing research with the actual situation of Wuhan, and regarded park characteristics, the built environment, and socioeconomic factors as the three kinds of influencing factors. Park characteristic factors can reflect the impact of individual park attributes on residents’ choices of park green space travel behavior. A section of the literature focuses on whether per capita park area [36], the number of park types [18,37], and the average distance to the nearest park [38] are associated with green justice. Built environment factors can reflect the surrounding situation of land utilization. Moreover, a large number of studies have focused on whether the differences in the availability of PGSs are caused by the differences in the built environment, such as per capita normalized difference vegetation index [39], the proportion of commercial land [40], the proportion of residential land [41], the proportion of industrial land [40], and the degree of land-use mix [42]. In addition, road network density [43] and the number of road intersections [42,44] can reflect the cost and convenience of getting to a destination. The built environment factors can provide a reference for transportation and land-use planning policy. Socioeconomic factors also reflect regional social development. Studies have verified that socioeconomic development plays an important role in the availability of PGSs to residents [8]. Urban central districts have a lower green space per capita due to their high population density [36], and green space per capita is selected to reflect the demand for green space. Gross domestic product [45], budget revenue of public finance [45], investment in fixed assets [46], and retail sales of consumer goods [47] represent the levels of economic development, government income, investment, and consumption, respectively, and significantly influence housing prices and living conditions [47,48]. Moreover, socioeconomic factors may provide a reference for population and social development policies. Table 1 shows the descriptive statistics of all selected variables.

### 2.3. Methods

#### 2.3.1. The Gaussian-Based 2SFCA Method for Accessibility

The 2SFCA model was first proposed by Radke [49] and further improved by Luo [50]. A number of scholars later conducted some extended studies on its limitations, which mainly involved the attenuation function [51], search radius [52], competition between supply and demand [53], multiple traffic modes [54], and expansion of youth groups [16]. Our study used the Gaussian-based 2SFCA model to measure the accessibility of different types of parks under walking and driving traffic modes. The Gaussian-based 2SFCA method can differentiate access to green space with integration of the Gaussian function and 2SFCA [55]. The standard of per capita park possession could be identified according to park types, and was used to determine park capacity in calculating accessibility. Based on the Code for Park Design (GB51192-2016) in China, the standards of per capita park area were as follows: comprehensive park as I (60 m^2^), theme park as II (50 m^2^), community park as III (40 m^2^), and amusement park as IV (30 m^2^). Therefore, the differences between different types of parks can be reflected in their accessibility. The park’s supply capacity coefficient *S_j_* is the weighted function of the maximum accommodating population and the function coefficient, and the formula is as follows:

(1) $$S_{j}=\frac{S_{j}{}^{A}}{pcpa_{j}}$$

where *S_j_^A^* is the area of each park, and *pcpa_j_* is the capacity standard of each park. According to the first step of the traditional 2SFCA model, the supply–demand ratio *R_j_* of each park within the predetermined time cost threshold is obtained, and the time distance is attenuated by the Gaussian function (*G*). In the second step, for each demand point *i*, we search all of the supply points (*j*) within the search radius (*t*_0_) of *i*. Meanwhile, Gaussian function attenuation is carried out again. Finally, we add all of the supply–demand ratios (*R_j_*) to obtain the accessibility *A_i_* as follows:

(2) $$A_{i}=\sum_{j\in \left\{t_{kj}\leq t_{0}\right\}}G\left(t_{kj},t_{0}\right)R_{j}=\sum_{j\in \left\{t_{kj}\leq t_{0}\right\}}G\left(t_{kj},t_{0}\right)\frac{S_{j}}{\sum_{k\in \left\{t_{ij}\leq t_{0}\right\}}G\left(t_{kj},t_{0}\right)P_{k}}$$

(3) $$G\left(t_{ij},t_{0}\right)=\{\begin{array}{cc} \frac{e^{-\left(1/2\right)\times {\left(t_{ij}/t_{0}\right)}^{2}}-e^{-\left(1/2\right)}}{1-e^{-\left(1/2\right)}}, & if,t_{kj}\leq t_{0}\\ 0, & if,t_{kj}>t_{0} \end{array}$$

In the wake of COVID-19, people have a new concept of “living community”, and the “15-min life circle” has become a lifestyle that most people yearn for. The 15-min community living circle is the basic unit to build community life. It is equipped with the corresponding basic service facilities and public activity space within the 15-min walking distance required by residents to form a healthy, safe, friendly, comfortable, and green social basic living platform [56]. Therefore, this paper takes 15 min as the search time threshold to compute the accessibility under walking and driving modes.

#### 2.3.2. The Gini Coefficient for Spatial Equity

The Gini coefficient was used as an indicator to evaluate the equity of green space. This is a common index used internationally to measure the income gap of residents in a country or region, as a single, simple, mathematical metric that represents the overall degree of inequality [6]. It is also a ratio of the area between the line of equality and the Lorenz curve divided by the total area under the line of equality [57]. The Gini coefficient ranges from 0 to 1—the maximum value is 1 and the minimum value is 0. In this case, 0 represents perfect equality of PGSs, while 1 represents perfect inequality, and as the coefficient approaches 0, the PGSs tend towards more equity. The mathematical calculation of the Gini coefficient is complex, but can be approximated using the following formula:

(4) $$G1=1-\overset{n}{\underset{k-1}{\sum }}\left(X_{k}-X_{k}-1\right)\left(Y_{k}+Y_{k}-1\right)$$

where *X_k_* is the cumulative proportion of the population variable for *k* = 0, …, *n*, with *X*_0_ = 0, *X_n_* = 1; and *Y_k_* is the cumulative proportion of the public transport service variable for *k* = 0, …, *n*, with *Y*_0_ = 0, *Y_n_* = 1.

#### 2.3.3. The Gradient Boosting Decision Tree (GBDT) Model

The GBDT model is a machine learning method integrating multiple weak classifiers, and its accuracy is higher than that of support-vector machines, random forests, and other algorithms in solving discrete classification problems with relatively concentrated data feature distribution [58]. Previous studies have shown that models based on decision tree integration can fit the influence of independent variables on dependent variables in different ranges instead of generating fixed coefficients, significantly improving the interpretability of the model [59]. The reliable identification of the relative importance and local relationships of variables by this model has been introduced into the field of urban transportation in recent years, such as trip choice [60] and trip distance prediction [61]. It also can output the potential nonlinear relationship diagram between independent variables and dependent variables [59], so as to better reveal the threshold effects of changes in the accessibility of urban parks. The multifold cross-validation method is used to reduce the influence of overfitting, and the local correlation graph generated by the model may produce abnormal noise values in the interval with few samples. We can combine the distribution of samples to weaken the unreliable estimation interval, and its approximation function *f*(*x*) of a group of independent variables *X* is the approximation function *f*(*x*) of multiple single decision trees $h\left(x;a_{m}\right)$:

(5) $$f\left(x\right)=\overset{M}{\underset{m=1}{\sum }}f_{m}\left(x\right)=\overset{M}{\underset{m=1}{\sum }}\beta_{m}h\left(x;a_{m}\right)$$

where *M* is the number of decision trees, $a_{m}$ is the residual coefficient of a single decision tree $h\left(x;a_{m}\right)$, and $\beta_{m}$ is the parameter minimizing the loss function L. The Gradient boosting algorithm, which is implemented as follows:

First, initialize the decision tree:

(6) $$f_{0}\left(x\right)={\mathrm{argmin}}_{\beta }\overset{N}{\underset{i=1}{\sum }}L\left(y_{i},\beta \right)$$

The total loss for all *N* samples is as follows:

(7) $$L_{all}=\overset{N}{\underset{i=1}{\sum }}L\left(y_{i},f_{m}\left(x_{i}\right)\right)$$

where $y_{i}$ and $f_{m}\left(x_{i}\right)$ are the actual value of sample $x_{i}$ and the predicted value of m models, respectively. For each iteration, its negative gradient function is as follows:

(8) $${\tilde{y}}_{im}=-{\left[\frac{\partial L\left(y_{i},f\left(x_{i}\right)\right)}{\partial f\left(x_{i}\right)}\right]}_{f=f_{m-1}}$$

The gradient descent step is calculated as follows:

(9) $$\beta_{m}={\mathrm{argmin}}_{\beta }\overset{N}{\underset{i=1}{\sum }}L\left(y_{i},f_{m-1}\left(x_{i}\right)+\beta h\left(x_{i};a_{m}\right)\right)$$

We can then update the calculation results to the model, and introduce the learning rate to control the contribution of each base tree, as follows:

(10) $$f_{m}\left(x\right)=f_{m-1}\left(x\right)+\xi \cdot \beta_{m}h\left(x;a_{m}\right),\;\mathrm{where}\;0<\xi \leq 1$$

The final model is as follows:

(11) $$f\left(x\right)=\overset{M}{\underset{m=1}{\sum }}f_{m}\left(x\right)$$

Better model results can be obtained by adjusting the hyperparameters in the model. A low learning rate can improve the prediction effect of the model, but more decision trees need to be added. In order to balance the running time and prediction performance, 5000 decision trees were selected, and the learning rate was set at 0.001. Moreover, a quintuple cross-validation method was introduced to reduce the influence of overfitting. The original dataset was divided into five parts, four different subsets were used for fitting in each iteration, and the remaining subsets were validated.

## 3. Results

### 3.1. Spatial Equality in Accessibility by Two Traffic Modes

#### 3.1.1. Spatial Accessibility for Two Traffic Modes

**Figure 2:**
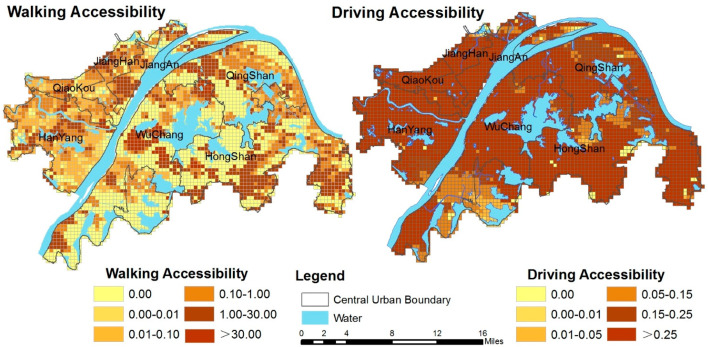
Spatial distribution of the accessibility of PGSs in the central urban area of Wuhan.

The accessibility of PGSs within 15 min via two traffic modes was analyzed using the 2SFCA method; Figure 2 shows the results. The areas with high walking accessibility are mainly located in eastern Hongshan, western Hanyang, Qiaokou, central Wuchang, and northern Qingshan, such as Yangchun Lake, the east of Yanxi Lake, and the Sand Lake. Areas with low accessibility are prominent in the south of Wuchang, the southwest of Hongshan, and the west of Yanxi Lake. More than 90% of the regions have high driving accessibility—especially the areas around the East Lake in Hongshan and both sides of the Yangtze River, such as the Hankou River beach and Wuchang River beach. In contrast, southern Wuchang, southwest Hongshan, and the east of Yanxi Lake have low accessibility.

#### 3.1.2. Spatial Equality in Accessibility

Table 2 shows that the inequality of accessibility by walking is much higher than that by driving throughout the whole region. The overall Gini coefficient of walking is as high as 0.96, and most of the districts—especially Hongshan and Qingshan—have obvious inequities. Although the overall Gini coefficient of driving is up to 0.51, most of the districts have higher equity (except for Hongshan), such as Hanyang and Qiaokou. Although the equity in some districts is not satisfactory, some of the streets have high equity. Therefore, the small-scale equity is also worth examining and discussing.

**Figure 3:**
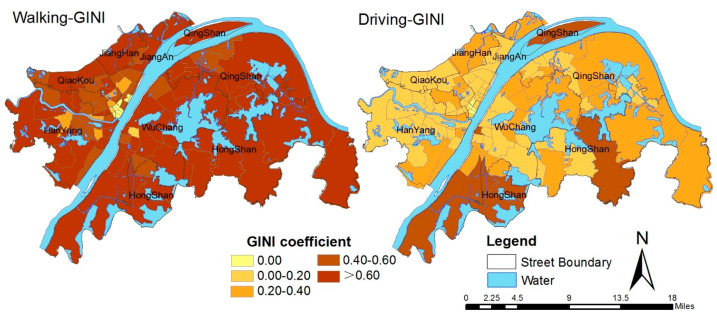
The Gini coefficients of accessibility and population by walking and driving.

Figure 3 illustrates the spatial equity of PGSs at the street scale. Significantly more areas have a high Gini coefficient in walking than in driving. Almost all of the streets of the urban center are areas with a high Gini coefficient by walking, except for a few streets in Jiangan, Jianghan, and Hongshan, such as Yiyuan Street, Taibei Street, and Shanghai Street. The Gini coefficient of most streets by driving is less than 0.4, which indicates that the inequity of residents’ enjoyment of PGSs is lower than that by walking. A few areas with high values are mainly distributed in the eastern and southern edges of Hongshan, which is consistent with the results of the Gini coefficient at the regional scale (see Table 3). Moreover, the areas with a low Gini coefficient under both modes are consistent, such as the east of Qiaokou and some streets in the north of Hanyang. The results suggest that some regions may show low equity when the demand and the supply of PGSs are relatively insufficient in the process of urban development, which may occur due to changes in population concentration, the improvement of road planning, and the increase in the number of parks. In addition, the overall equity of the district is not entirely consistent with the street level, and a possible reason for this is the inconsistency of the increase in the range of the community population, PGSs, and roads. The specific reasons need to be further explored.

### 3.2. Nonlinear Influence on Accessibility and Equity

#### 3.2.1. Analysis of the Relative Importance of Influencing Factors

By comparing the results of the OLS and GBDT models, we found that the R^2^ values in OLS are lower than those in GBDT, indicating that the feasibility of OLS is low and the linear relationships between the variables are weak. Therefore, the GBDT model was adopted to explore the nonlinear effects of variables on accessibility and equity. Table 3 illustrates the relative importance and ranking of the independent variables. The sum of the relative importance of all influencing factors is 100%.

PD had the largest contribution (20.86%) to the accessibility of PGS by walking, followed by IFA (17.35%), PI (14.43%), GDP (13.75%), and RCG (12.87%). These five variables explain more than half of the predicted results, whereas the importance of the other variables is similar, except for that of PTN (0.04%). PD also had the most significant contribution (25.35%) to driving accessibility, followed by P-NDVI (23.15%), PR (12.14%), PC (8.01%), and RD (7.46%), whereas the significance of other variables to PGS was at similar levels, with weak explanatory power. Overall, socioeconomic and built environment factors contributed the most to the accessibility by walking and driving, respectively, with good explanatory power.

PR had the highest contribution (34.73%) to the equity of PGS by walking, followed by SAD (7.70%), whereas the contribution of PTN was not significant. SAD (10.76%) had the greatest contribution to the equity by driving, followed by PI (10.01%) and PR (9.81%), whereas PTN had the lowest contribution. PR and SAD contributed the most to the equity and accessibility by walking, with good explanatory value, while the built environment had the most obvious influence on the equity by driving.

#### 3.2.2. Threshold Effect of Key Variables in Accessibility

**Figure 4:**
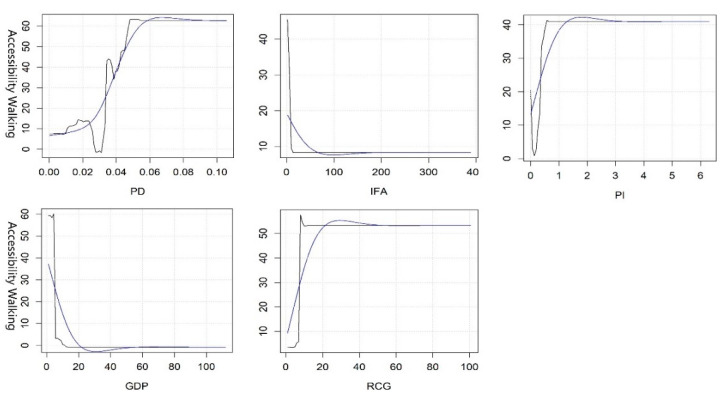
Nonlinear effects of various variables on the accessibility of PGSs by walking.

On the basis of the results of the GBDT model, we chose to analyze the key variables whose rate of contribution was more than 7%. Since the original curve is too complex and has too many details, we only need to know the approximate threshold range to make reference for planning. Therefore, we use the smooth blue curve for non-linear analysis. As shown in Figure 4, the PI, PD, IFA, GDP, and RCG are the five key variables affecting accessibility by walking. When the PI reaches 1.5, the accessibility is the best. The impact on accessibility is not significant when it reaches 3. PD is positively correlated with accessibility to a certain extent. When it reaches 0.07, residents can enjoy the highest accessibility, but it has almost no influence on accessibility when it is greater than 0.08. Both IFA and GDP are negatively correlated with accessibility, and the accessibility is the worst when the IFA reaches 1 million/person and GDP reaches 0.3 million/person. With the increase in RCG to 0.3 million/person, accessibility reaches the highest, and the accessibility is relatively stable when it increases to 0.5 million/person.

**Figure 5:**
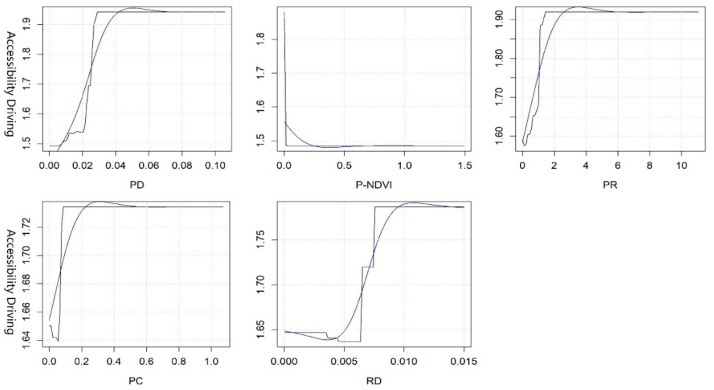
Nonlinear effects of various variables on the accessibility of PGSs by driving.

The variables P-NDVI, PR, PC, RD, and PD are the five key variables affecting the driving accessibility (see Figure 5). Among the built environment factors, P-NDVI is negatively correlated with accessibility to a certain extent. When P-NDVI reaches 0.4, the accessibility is close to 1.5, whereas accessibility is almost unaffected when the P-NDVI reaches 0.5. PR, PC, and RD are positively correlated with accessibility to some extent. The interpretability of the model is good when the PR increases to 0.35, PC increases to 0.3, and RD increases to 0.011, showing that residents can enjoy the best PGS service. However, accessibility is relatively stable when they exceed the threshold. Among the socioeconomic factors, PD is positively correlated with accessibility, and residents can enjoy the highest accessibility when it reaches 0.05.

#### 3.2.3. Threshold Effects of Key Variables in Equity

**Figure 6:**
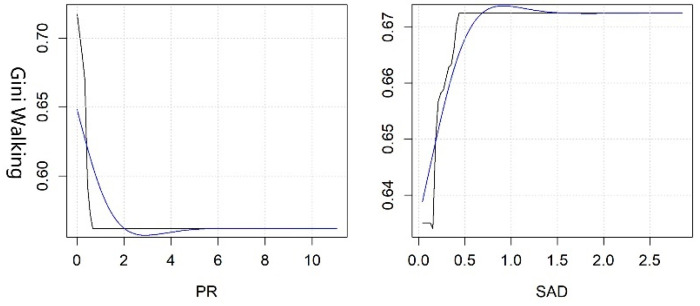
Nonlinear effects of various variables on the equity of PGSs by walking.

The SAD of the park and the PR of the built environment are two key variables affecting the equity by walking, and their rate of contribution accounts for nearly half (see Figure 6). When the SAD is up to 0.1 km, residents can enjoy the greatest equity, while inequity is most prominent when the SAD is 0.9 km; when the SAD is more than 1.5 km, the explanatory power of the model is poor. The PR is negatively correlated with equity. When it reaches 3, the model can be interpreted well, which means that residents can only enjoy limited equity of PGSs; the equity is relatively stable when it exceeds 5.

**Figure 7:**
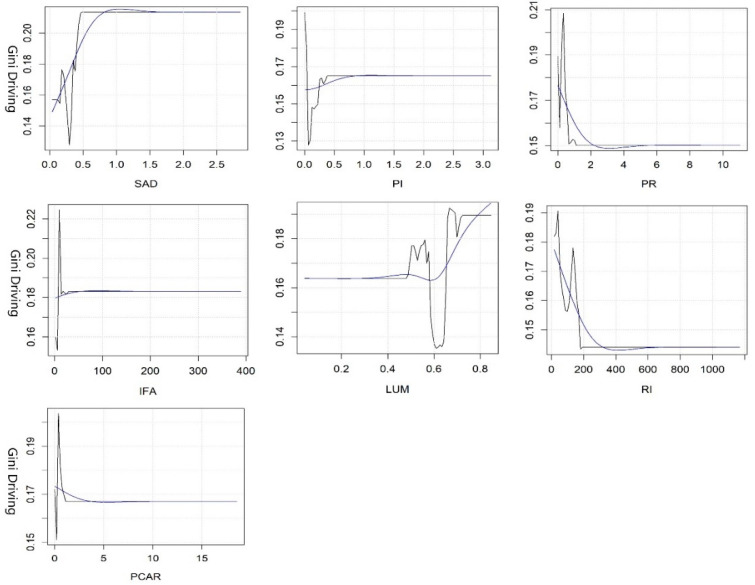
Nonlinear effects of various variables on the equity of PGSs by driving.

The three kinds of variables have different degrees of influence on the equity by driving—especially the PCAR, SAD, PI, PR, LUM, RI, and IFA (see Figure 7). Equity improves when the PCAR increases to 4 m^2^/person, and equity is relatively stable when it exceeds the threshold. Moreover, inequity is highest when the SAD reaches 1 km. When the PI reaches its minimum, which is close to 0, the equity is the best. However, the degree of equity gradually decreases and tends to be stable when it reaches 0.9. Residents can enjoy the best equity when the PR reaches 3, and the inequality rises gradually and tends to be stable beyond this threshold. When the LUM reaches about 0.6, the inequity of residents’ enjoyment of park services is the worst. The inequity shows an obvious upward trend when the mixed use of land increases to more than 0.6. When the RI increases to 400, the equity constantly improves; the equity is not significantly affected when it exceeds 600. The increase in IFA has a relatively stable impact on the equity of PGSs.

## 4. Discussion

### 4.1. Advantages of the GBDT Method for Green Justice

This study explored a new nonlinear relationship between equity in accessibility and multidimensional factors, which could rank the importance of these factors. Our findings suggest that park characteristics [62], the built environment [10], and socioeconomic factors [30,32,47] present different impacts on the spatial accessibility and equity of PGSs, which is consistent with the findings of previous studies. We identified key variables and analyzed their synergistic and heterogeneous effects on accessibility and equity, which was the difference between our study and others. Heterogeneity exists in the key variables, thus affecting accessibility and equity. The improvement of accessibility may not alleviate inequity, and the improvement of equity may not improve accessibility.

Comparing OLS with the GBDT model, our study further verified the advantages of the GBDT model, in that it can reduce the influence of outliers, deal with multicollinearity problems, and provide more accurate prediction results [63]. As the provision of green space is mostly unevenly distributed, and tends to be spatially heterogeneous [3], simple statistical methods such as global ordinary least squares (OLS) regression may not be applicable. The homoscedasticity assumption of OLS may be violated and only produce space-constant global relationships that only reflect the average conditions [32]. Nonetheless, several variables have a significant impact on the accessibility and equity under the linear model. For example, previous studies have confirmed the relationship between the distance and efficiency of park utilization [17,64]. Specifically, residents with a distance of 100 m are more willing to go to the park than residents who live more than 100 m from the park [65]. Our study also found that only RCG and SAD had significant effects on the accessibility by walking and the equity by driving on the basis of OLS models (see Table 3). However, the threshold effects on the accessibility and equity of PGSs, which may guide planning practice, were not revealed. Studies suggest that the accessibility of urban green space is low in communities with low levels of socioeconomic development [32], but the threshold of economically disadvantaged communities causing low accessibility is unclear. If the thresholds of key influencing variables can be clearly defined, then urban planning and fine governance can be well guided. The GBDT model may be an appropriate method. This model can not only deal with the multicollinearity problem, but also outputs the potential nonlinear relationship between independent variables and dependent variables, so as to reveal the threshold effect of changes in the accessibility of urban park green spaces. Moreover, the nonlinear influencing variables can be ranked by relative importance, which can guide the order of policy regulation.

**Figure 8:**
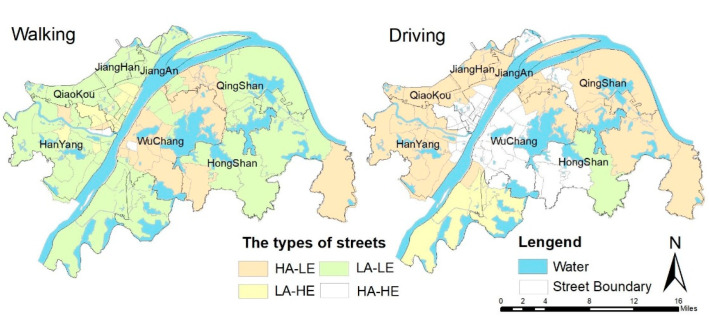
Street distribution of three types by walking and driving.

Our applications and findings of the GBDT method bring three contributions to existing research: First, accessibility and equity were used to evaluate green justice comprehensively. Previous studies have tended to take the center of the street as the origin when measuring street accessibility, which is not consistent with reality [66]. This study measured accessibility on the basis of grid scale, and calculated the average accessibility of streets. The Gini coefficient was used to measure the spatial equity of streets and regions, providing guidance for urban zoning and facilitating fine governance. Second, our study verified that the GBDT model could better reveal the threshold effect of PGS equity compared with traditional linear models using OLS. Third, our research focused more on areas where accessibility and equity levels differ, and identified thresholds of key variables in different scenarios. By adjusting the thresholds of key variables, urban planners can achieve different regulatory objectives. For example, the HA-LE areas should give priority to relevant policy adjustments that promote key variables of equity (see Figure 8), indicating better planning practice that does more than emphasize the reconstruction of large parks far from residential areas, as discussed in previous studies [34].

### 4.2. Implications for Urban Planning Based on the Effects of Heterogeneity and Synergy

Our findings indicate that the key variables affecting spatial accessibility and equity show strong synergistic and heterogeneous effects. In order to guide the planning practice, we constructed four scenarios corresponding to the street unit for realistic park services (see Figure 8): high accessibility with low equity (HA-LE), low accessibility with high equity (LA-HE), low accessibility with low equity (LA-LE), and high accessibility with high equity (HA-HE). Walking accessibility greater than 10 and driving accessibility greater than 1 is regarded as high accessibility, and a Gini coefficient greater than 0.5 by the two modes is considered low equity. The HA-HE areas by driving are mainly located in the core areas of Wuhan’s urban center, but these areas are limited in terms of walking. These areas showed that the key variables reached the threshold and presented synergistic effects. The LA-LE areas by walking are mainly distributed in streets with obvious low accessibility and low equity, except for the areas around the Sand Lake and East Lake. The low accessibility is mainly because the values of key variables—such as PI, PD, IFA, GDP, and RCG—are lower or higher than the optimal threshold. The main reason for low equity was the low proportion of SAD and PR. The HA-LE areas by walking are mainly located in Wuchang’s core area and some streets of Hongshan and Qiaokou, and only in Jiufeng Township by driving. The priority should be given to key variables that have a relatively significant impact on the equity of PGSs. The LA-HE areas are located in Jiangan and Qiaokou for walking and the south of Hongshan for driving. Emphasis should be placed on key variables that may have a relatively significant impact on accessibility, thereby facilitating improvements in accessibility.

It can be seen that key variables in different modes have different nonlinear effects on accessibility and equity, and sometimes the same variable has opposite effects, which may be the result of the synergistic effects of different factors [67]. Therefore, the synergistic effects of key variables should be considered in the LA-LE and HA-HE areas by adjusting the same variables. The HA-LE and LA-HE areas need to adjust the key variables by heterogeneous effects according to the planning target, and targeted and specific urban planning implications should be selected for regions with different scenarios for similar cities.

First, policy strategies should be given priority in HA-LE areas (see Figure 8), including land-use and transportation policies. The resistance of the shortest distance between a residential area and the nearest park should be taken into account, and it is best to shorten the average distance as much as possible (to about 100 m). Industrial land should be appropriately reduced near residential areas and green park areas. Meanwhile, the number of road intersections affects residents’ travel time [68]; it should be reasonably increased in the streets, and the total number ought to be at least 400 to improve road connectivity, shorten the time need for residents to reach the park, and improve equity.

Second, the PD, SAD, PI, PR, IFA, LUM, and RI are key variables in walking and driving. Therefore, land use, transportation, population, and economic policies play an important role in LA-HE areas. The population density of residential areas can be appropriately increased so that the population can reach a balance between supply and demand within a reasonable range, which may ensure that the parks are readily accessible to residents [69]. A rise in RCG means an increase in consumer demand, which may improve residents’ income, and income level has a significant influence the choice of travel behavior. It is important to improve RCG and promote consumption, which can lead to the improvement of accessibility. Appropriate adjustments should also be made to the proportion and rational allocation of land use in streets [30]. For example, the PR and PC should be increased to about 0.3. In addition, residential roads in streets should be rationally planned to reduce the occurrence of dead-end roads.

Third, the synergistic effects of key variables on accessibility and equity should be given more priority in LA-LE areas. In other words, priority should be given to key variables that have an impact on both accessibility and equity, while simultaneously adjusting policies and programs related to these key variables to reach reasonable thresholds. Then, other key variables should be gradually adjusted according to local conditions. In Wuhan, the government should pay more attention to the inputs of economy in these areas and the optimization of the built environment to improve the accessibility and equity, such as the PI, PD, PR, IFA, GDP, RCG, etc. Among these variables, PR is the key variable of priority adjustment, and its optimal threshold is close to 1 (see Figure 4, Figure 5, Figure 6 and Figure 7).

### 4.3. Limitations

However, some limitations could be improved in future studies. First, we ignored the differences in demand and preferences of different groups in enjoying park resources—for example, the elderly tend to prefer the nearest park. Studies have shown that individual behavioral intentions may have an important impact on accessibility [70]. Second, we only considered the equity of green spaces by two modes of transport—walking and driving—but other modes of transportation, such as cycling and public transportation, should not be ignored. Most previous studies have shown that different traffic modes result in different spatial accessibility [5]. Moreover, the dynamics of green space equity and the influencing factors driving these changes were not considered in our study [47]. Therefore, future studies could measure the accessibility and equity of PGSs based on the attributes of different groups and other modes of transportation, and could try to study the spatiotemporal changes and driving factors of PGSs from a process perspective.

## 5. Conclusions

This study applied the GBDT model to investigate the nonlinear relationships between multiple variables and equity in accessibility in Wuhan’s central districts. Our findings offer several insights for urban green space planning and urban health. The analysis of the spatial distribution differences of accessibility and equity of PGSs enables us to have a better understanding of green justice in urban areas. Accessibility and Gini coefficients can be used as the effective indicators to evaluate green justice. The nonlinear regression analysis through the GBDT model was used to discuss the influencing factors of accessibility and equity, and helped us to highlight the relative importance of key variables as well as their synergies and differences, showing the advantages of the nonlinear model. Moreover, we classified the streets as different combination types (i.e., HA-LE, LA-HE, LA-LE, and HA-HE) according their accessibility and equity. We found that accessibility or equity should be improved for different types of streets, and offered specific planning suggestions, making our analysis clear and concrete. These findings demonstrate the importance of the nonlinear analysis and threshold effects in the GBDT model to provide specific guidance and effective strategies for green space decision-makers and planners. Future studies could explore other themes—such as green space demand, travel behavior prediction, public health, and environmental justice—by taking advantage of the GBDT model.

## Figures and Tables

**Table 1:** Descriptive statistics of all variables.

Variables	Description	Mean (St. Dev)
**Park characteristic factors**		
Per capita park area (PCAR)	Per capita park area of each street	0.994 (2.596)
Park type number (PTN)	Park Type number of each street	2 (1)
The shortest average distance to the nearest park (SAD)	The shortest average distance to nearest park in each street	0.318 (0.365)
**Built environment factors**		
Per capita normalized difference vegetation index (P-NDVI)	Per capita NDVI of each street	0.091 (0.264)
Road network density (RD)	The ratio of total road length to street area	0.005 (0.003)
Road intersections (RI)	Number of road junctions in each street	116 (176)
Proportion of commercial land (PC)	The ratio of commercial land area to street area	0.108 (0.182)
Proportion of residential land (PR)	The ratio of residential land area to street area	0.398 (0.956)
Proportion of industrial land (PI)	The ratio of industrial land area to street area	0.904 (1.744)
Degree of land-use mix (LUM)	Diversity of land-use types in each street	0.587 (0.158)
**Socioeconomic factors**		
Population density (PD)	The ratio of population to street area	0.026 (0.024)
Gross domestic product (GDP)	Sum of the added value of various industries in each street	11.578 (18.257)
Retail sales of consumer goods (RCG)	The sum of retail sales of consumer goods to urban and rural residents and social groups	9.210 (14.263)
Budget revenue of public finance (PFBR)	Tax and non-tax revenue independently used by fiscal authorities	0.790 (1.222)
Investment in fixed assets (IFA)	A comprehensive index reflecting the relationship between the scale, speed, and proportion of investment in fixed assets	20.565 (45.772)

**Table 2:** Gini coefficients of different districts under two traffic modes.

Districts Name	Grid Numbers	Gini—Walking	Gini—Driving
Jiangan	792	0.71	0.38
Jianghan	609	0.82	0.44
Qiaokou	107	0.93	0.37
Wuchang	181	0.92	0.43
Hongshan	167	0.98	0.54
Qingshan	1244	0.93	0.38
Hanyang	568	0.88	0.29
Overall	3684	0.96	0.51

**Table 3:** Relative importance of three categories of influencing factors on the service equity of PGSs (%).

Variables	OLS Model			GBDT Model (Rank/Relative Importance (%))			
	AI—Driving	Gini—Walking	Gini—Driving	AI—Walking	AI—Driving	Gini—Walking	Gini—Driving
**Park characteristic factors** (**the sum of all relative importance**)				1.92	5.42	14.45	20.24
PCAR	0.029	−0.003	−0.006	(10) 1.88	(9) 2.84	(4) 6.21	(7) 7.53
PTN	−0.008	0.098	0.003	(15) 0.04	(14) 0.04	(15) 0.54	(15) 1.95
SAD	−0.285	0.308	0.118	(7) 4.18	(12) 2.53	(2) 7.70	(1) 10.76
**Built environment factors** (**the sum of all relative importance**)				28.56	60	67.25	51.76
P-NDVI	−0.432	0.062	0.233	(6) 4.74	(2) 23.13	(8) 5.47	(12) 4.75
RD	91.090	−46.123	−0.834	(14) 0.45	(5) 7.46	(7) 5.57	(10) 5.41
RI	0.000	0.003	0.000	(11) 1.88	(8) 2.90	(6) 5.80	(6) 7.96
PC	0.370	−0.014	−0.069	(9) 2.32	(4) 8.01	(3) 6.68	(11) 5.29
PR	−0.069	0.002	0.004	(12) 1.32	(3) 12.14	(1) 34.73	(3) 9.81
PI	0.219	0.069	−0.012	(3) 14.43	(11) 2.59	(10) 4.21	(2) 10.01
LUM	−1.591	0.612	0.202	(8) 3.42	(7) 3.77	(9) 4.79	(5) 8.44
**Socioeconomic factors** (**the sum of all relative importance**)				65.34	34.56	18.28	28.08
PD	22.294	−0.271	−1.205	(1) 20.86	(1) 25.35	(5) 6.08	(13) 3.90
GDP	−0.185	−0.036	0.000	(4) 13.75	(13) 1.06	(11) 3.96	(8) 6.76
RCG	0.032	0.005	0.002	(5) 12.87	(6) 5.27	(13) 3.32	(9) 5.93
PFBR	2.195	0.458	−0.071	(13) 0.51	(15) 0.20	(14) 1.44	(14) 3.03
IFA	0.008	0.002	0.000	(2) 17.35	(10) 2.68	(12) 3.48	(4) 8.46
R^2^	0.26	0.39	0.30	0.32	0.42	0.57	0.92

## Data Availability

Not applicable.
